# Supplementary material for: MUC5B Polymorphism in Patients with Idiopathic Pulmonary Fibrosis—Does It Really Matter?
Source: Int J Mol Sci. 2025 Feb 28;26(5):2218. doi: 10.3390/ijms26052218 (PMC11900561; doi:10.3390/ijms26052218)
Supplement: Supplementary file 1 [file ijms-26-02218-s001.zip › ijms-3480126-supplementary.pdf]

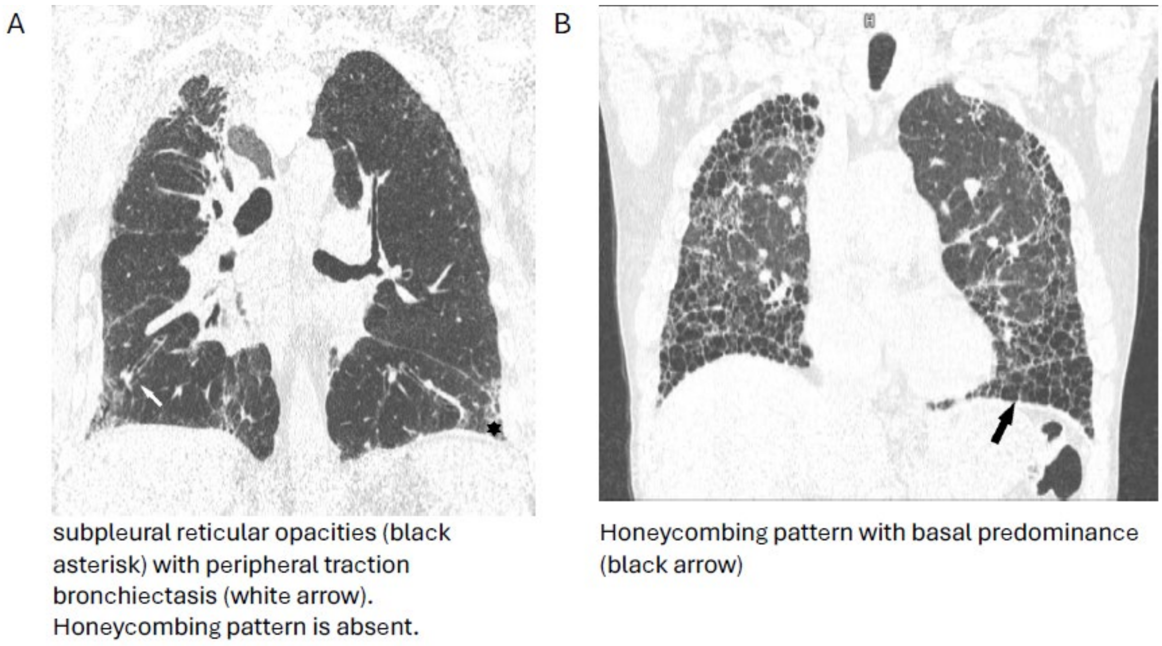

Figure S1. HRCT scans of patients with probable UIP (**A**) and UIP (**B**) pat-terns—coronal presentation.

Table S1. Comparison between the incident and prevalent cases.

	**Prevalent cases (n=47)**	**Incident cases (n=46)**	**P value**
Sex			
**Males, n (%)**	**36 (76.6)**	**24 (52.2)**	**0.0176**
**Age at dgn, yrs (mean±SD)**	**66.4±7.1**	**71.45±8.2**	**0.0021**
Smoking status, n (%)			
never	5 (10.6)	10 (21.7)	0.1688
former	42 (89.4)	36 (78.3)	
active			
Packyears, median (IQR)	30 (15 – 50)	23.5(15-43.75)	0.3884
**Length of follow-up, mths (mean±SD)**	**91.7±28.9**	**47.9±18.7**	**<0.0001**
Baseline FVC, %pred (mean±SD)	90.6±18.5	89.5±18.5	0.7756
Baseline TL,co, %pred (mean±SD)	59.3±15.6	58.3±15.75	0.7479
Baseline 6MWD, m (mean±SD)	482.1±95	478.9±101.8	0.8835
Baseline desaturation, % (median, IQR)	3 (1 – 6)	4 (1 – 8.25)	0.6650
GAP index, n (%)			
I	35 (74.5)	32 (71.1)	0.8160
II	12 (25.5)	13 (28.9)	
III	0	1	
HRCT (n=92)			
**UIP**	**42 (89.4)**	**32 (71.1)**	**0.036**
**Probable UIP**	**5 (10.6)**	**13(28.9)**	
Antifibrotic treatment, n (%)	46 (97.9)	42 (89.4)	
Pirfenidone	24 (52.2)	19 (45.2)	0.8
Nintedanib	13 (28.3)	16 (38.1)	
Both (consecutively)	9 (19.5)	7 (16.7)	
**Time to treatment initiation (months), n=88 (median, IQR)**	**34.4 (17.9 – 55.8)**	**6.95 (1.1 – 15.7)**	**<0.0001**
Length of treatment, m (mean±SD)	**53.5**±21.3	**36.8**±**13.5**	**<0.0001**
Lung cancer, n (%)	8 (17)	5 (10.8)	0.5516
**Death/transplant, n (%)**	**29 (63)**	**15 (34)**	**0.0036**
MUC5B status, n (%)			
GG	17 (36.2)	11 (23.9)	0.1976
GT	27 (57.4)	32 (69.6)	
TT	3 (6.4)	3 (6.5)	
MAF, %	35.1	41.3	0.4508

FVC—forced vital capacity, TL,co—transfer factor of the lungs for carbon monoxide, GAP—Gender–Age–Physiology Index, HRCT—high-resolution computed tomography, UIP—usual interstitial pneumonia, SD—standard deviation, IQR—interquartile range, MAF—minor allele frequency.
